# The Effect of Chokeberry Juice and Fiber Consumption on the Concentration of Antioxidant Minerals in Serum

**DOI:** 10.3390/antiox14050516

**Published:** 2025-04-25

**Authors:** Ewa Olechno, Małgorzata Elżbieta Zujko, Katarzyna Socha, Anna Puścion-Jakubik

**Affiliations:** 1 Department of Food Biotechnology, Faculty of Health Science, Medical University of Białystok, Szpitalna 37 Street, 15-295 Białystok, Poland; ewa.olechno@sd.umb.edu.pl (E.O.); malgorzata.zujko@umb.edu.pl (M.E.Z.); 2 Department of Bromatology, Faculty of Pharmacy with the Division of Laboratory Medicine, Medical University of Białystok, Mickiewicza 2D Street, 15-222 Białystok, Poland; katarzyna.socha@umb.edu.pl

**Keywords:** minerals, chokeberry juice, chokeberry fiber, serum

## Abstract

Aronia is a fruit that has become increasingly popular in recent years. Numerous health-promoting effects resulting from consumption have been indicated, including the possibility of using aronia as a functional food in the prevention and support of therapy for various ailments. For the first time, we assessed the effects of the impact of a 90-day nutritional intervention on the level of selected minerals in the serum of women (*n* = 67) and men (*n* = 35). The consumption of 100 mL of juice for 60 days resulted in a significant increase in the levels of copper, manganese, and selenium. The addition of 10 g of aronia fiber resulted in a further increase in the concentration of these elements with antioxidant properties. An interesting observation is that the concentration of zinc decreased, which may indicate the operation of compensatory mechanisms. The above results indicate that regular consumption of aronia bio-products may have a beneficial effect on the concentration of microelements in the serum.

## 1. Introduction

Chokeberry fruits are known for their health-promoting properties. Their hypoglycemic, hypolipidemic, hypotensive, and hepatoprotective effects have been demonstrated [1,2,3]. Our previous publication has also shown that a nutritional intervention based on chokeberry products—chokeberry juice and fiber—has a beneficial effect on selected metabolic parameters. After 90 days of intervention with chokeberry juice, statistically significant increases in the antioxidant potential of the serum were observed. A decrease in other parameters was also observed, including the waist circumference, systolic blood pressure, diastolic blood pressure, heart rate, glycated hemoglobin, glucose, LDL cholesterol, eGFR, and ALT level [4].

The rich biochemical composition of chokeberry fruits is responsible for these effects. They contain many compounds, including polyphenols and vitamins, among others vitamin C, vitamin K and vitamin E, carotenoids, macroelements like magnesium (Mg) and trace elements such as copper (Cu), iron (Fe), manganese (Mn), selenium (Se), and zinc (Zn) [5,6]. Chokeberry fruits have a particularly high antioxidant potential compared to other fruits [7]. Researchers underline the role of polyphenols [6,8]; however, it should be emphasized that other components of chokeberry may also be important. The qualitative assessment of chokeberry products—juices and fiber—allowed us to state that these products can be defined as a source of important selected minerals, including antioxidant elements such as Zn, Se, Mn, Cu, and Fe [5]. These elements are involved in fighting reactive oxygen species [9]. They are components of antioxidant enzymes and their activators or cofactors, and their action helps maintain the antioxidant–oxidant balance of the body [10]. Oxidative stress is associated with an excessive production of reactive oxygen and/or nitrogen species and, at the same time, with a deficiency of antioxidants, both enzymatic and non-enzymatic. The body naturally produces pro-oxidants; however, their excess relative to the antioxidant defense mechanisms can lead to adverse effects. Excess reactive oxygen/nitrogen species, in turn, contribute to cellular damage—DNA, proteins, and lipids. These damages can, in turn, lead to numerous pathological conditions, such as neurodegenerative diseases, circulatory system diseases, diabetes and kidney diseases, and cancer [11]. A properly balanced diet, rich in antioxidants, can help to prevent and treat chronic diseases [12,13,14,15]. Human demand for microelements is less than 100 mg per day, but they play a necessary role in maintaining health [16].

Zn is involved in the activation of over 300 enzymes. It plays a role in the proper functioning of the sense of smell and taste, has a positive effect on insulin sensitivity, fertility, and the proper functioning of the reproductive and immune systems [17]. This micronutrient helps maintain the antioxidant–oxidant balance by inducing metallothionein [18,19]. Additionally, it competes with Fe and Cu, which, in excess, have pro-oxidant properties [20]. This element also inhibits the pro-oxidant enzyme nicotinamide adenine dinucleotide phosphate oxidase (NADPH-oxidase), is a cofactor of many antioxidant enzymes, and is also part of the enzyme superoxide dismutase (Zn/Cu SOD), which is responsible for combating superoxide radicals [21,22].

Cu occurs in the body in two forms—reduced form (Cu I) or oxidized form (Cu II). This element acts as a cofactor of antioxidant enzymes, including cytochrome C oxidase, superoxide dismutase, lysyl oxidase, and tyrosinase [22]. Cu catalyzes the oxidation of Fe as a component of ceruloplasmin [23]. It is involved in the production of energy in cells, the formation of hemoglobin, myelin, and the formation of thyroid hormones [24,25].

One of the indicators of health status may be the Cu/Zn ratio. A high Cu/Zn ratio is associated with chronic inflammatory diseases [26], malnutrition [27], increased oxidative stress, inflammation, and impaired immune status in patients with chronic diseases [28]. The Cu/Zn ratio indicates the interaction between these two trace elements and is a stronger predictor of many pathologies than individual Zn and Cu levels [29].

Mn is the main component and activator of pyruvate carboxylase, glycosyltransferase, glutamine synthetase, alkaline phosphatase, and mitochondrial superoxide dismutase [30,31]. This mineral ingredient occurs in tea, nuts, legumes, cereal products, seafood, and to a lesser extent in some vegetables and fruits [32].

Se in the body exerts its effects mainly through its incorporation into selenoproteins [33]. So far, 25 selenoproteins have been discovered in the human body, including the glutathione peroxidases [34]. Selenoproteins are associated with many metabolic and functional pathways, such as infections, aging, and cancer [35]. As a cofactor of enzymes involved in antioxidant protection, Se may play a role in regulating various inflammatory processes in the body [36].

As shown in our study, chokeberry juice and fiber may be a good source of microelements in the diet [5]. This prompted us to conduct further studies aimed at assessing the consumption of chokeberry juice and fiber on the concentration of selected minerals in serum. The aim of this study was to assess the impact of a dietary intervention using organic chokeberry products (chokeberry juice and chokeberry fiber) on the level of some antioxidant elements in the serum. To our knowledge, the impact of the consumption of chokeberry products on the content of antioxidant minerals in serum has not been studied so far.

## 2. Materials and Methods

### 2.1. Participants

This study was conducted among residents of Poland. Volunteer participants expressed their willingness to participate in the study by completing the online application form (using Google Forms). The inclusion criteria for the study were ages 30–65 and low or moderate physical activity levels. The following exclusion criteria were adopted at the study design stage: diabetes, selected gastrointestinal diseases (intestinal diseases, including inflammatory bowel diseases and functional bowel disorders, as well as stomach ulcers, chronic or acute gastritis), taking medications that lower lipid levels and glucose levels, immunosuppressive, anticoagulant or antihypertensive drugs, use of steroid therapy; high physical activity, pregnancy, and breastfeeding. After we considered the data indicated in the surveys and analyzed the exclusion criteria, 102 people (67 women and 35 men, 30–65 years) were classified to participate in the study.

The protocol of the study was approved by the Local Bioethical Committee. Written consent was obtained from all participants.

### 2.2. Dietary Intervention

The participants were asked to maintain the same diet in each of the three stages to avoid interference from other dietary components. Dietary data (caloric intake, protein intake, fat intake, and carbohydrate intake) are presented in our previous study. Participants completed dietary recall interviews on 2 workdays and 1 weekend day to determine whether the dietary intake of individual nutrients, including polyphenols, was stable at each stage of the study, the results of which are presented in Table 1 [4]. During the intervention, participants consumed 100 mL of chokeberry juice every day (for 60 days), after which they additionally consumed 10 g of chokeberry fiber (for 30 days). The products for the intervention were selected based on our previous studies—they had the most beneficial composition, and the portion of the product was to cover the demand for ingredients with antioxidant properties to the greatest extent. The biochemical composition of the chokeberry juice and fiber used is presented in Table 2.

Participants could dilute the juice with spring, filtered, or boiled water. The chokeberry fiber could be divided into two portions a day (5 g of fiber per serving) and consumed with water or included in a meal. Participants were asked to discontinue the use of antioxidant supplements for at least 7 days prior to laboratory testing and for the duration of the dietary intervention. Additionally, they were advised not to consume larger amounts of chokeberry juice per day than recommended, as well as other juices containing chokeberry, supplements, or other products containing chokeberry fruit.

A determination of mineral content was performed three times during the study: before the start of the dietary intervention (stage 0), after 2 months of consuming chokeberry juice (60 days, stage 1), and after 3 months (next 30 days, stage 2), i.e., after the monthly consumption of chokeberry juice and fiber together. 

Our previous studies have shown that one serving of chokeberry juice (100 mL) can cover the demand for Cu in the range from 149.5 to 3177.0%, for Mn: from 6.8 to 32.4%, for Se: from 0.9 to 7.4%, and for Zn: from 0.3 to 1.2%. Fiber, which was used during the intervention to enhance the effect of the juice, covered the demand for Cu in the amount of 3.7 to 8.2%, for Mn: 15.8 to 20.3%, for Se: 0.6 to 9.0%, and for Zn: 0.9 to 8.5% [5].

### 2.3. Determination of Serum Mineral Content

Blood samples of approximately 6 mL were collected using vacutainer test tubes containing clot activator (Becton Dickinson, Franklin Lakes, NJ, USA. Blood was centrifuged for 10 min (approximately 1000× *g*), sera were collected and stored at −20 °C. Serum samples were deproteinized with 1 mol/L spectral grade nitric acid (Merck, Darmstadt, Germany), and 1% Triton X-100 was added, then mixed via vortex and centrifuged (10 min).

Serum concentrations of antioxidant elements were determined using electrothermal (Cu, Mn, and Se) and flame (Zn) atomic absorption spectrometry with Zeeman background correction at wavelengths of 324.8 nm, 279.5 nm, 196.0 nm, and 213.9 nm, respectively (Z-2000 apparatus, Hitachi, Tokyo, Japan). Working solutions were prepared from 1 g/L standard solutions (Merck, Darmstadt, Germany). A certified reference material of human serum matrix (Seronorm Trace Elements, Serum Level 1, 0903106, Sero AS, Billingstad, Norway) was used to assess the accuracy of the method. All results of control samples corresponded to the reference values, and the precision scores were as follows: 2.4% for Cu, 3.3% for Mn, 3.2% for Se, and 1.9% for Zn. Additionally, the Cu:Zn molar ratio was calculated.

The determined concentrations of mineral components in serum were interpreted in relation to the reference values as follows: 0.7–1.6 mg/L for Cu, 0.2 to 1.1 µg/L for Mn, 66–104 µg/L for Se, and 0.7–1.3 mg/L for Zn [37]. To check the accuracy of the method, an internal standard was added.

### 2.4. Statistical Analysis

The results regarding the concentration of elements in serum were developed using two programs: Microsoft Office Excel 2019 and Statistica 13.3 (StatSoft, Tibco, Palo Alto, CA, USA).

The following parameters of descriptive statistics were obtained: average (Av.) with standard deviation (SD), minimum and maximum (Min. and Max.), median (Med.), and lower and upper quartiles (Q1 and Q3). The normality of the distribution of numerical data was assessed using the following tests: Kolmogorov–Smirnov, Lilliefors, and Shapiro–Wilk tests.

The Wilcoxon signed-rank test was used to assess differences between individual groups before and after the intervention. The Spearman correlation assessment allowed determining the strength of relationships between the studied parameters. The statistical significance level was assumed at *p* < 0.05.

## 3. Results

The results of our study are presented in Table 1, Table 2, Table 3 and Table 4.

Table 1 presents the intake of selected minerals and vitamins, as well as the FRAP value, at different stages of the study, namely, at the beginning of the intervention, after the stage in which participants consumed juice only, as well as at the end of the experiment. The intake of selected nutrients at individual stages did not differ with any statistical significance.

Table 2 presents the characteristics of chokeberry juice and fiber. The data indicate that the FRAP value for the juice was significantly higher (97.41 vs. 6.51 mmol/L). The TPC for the juice was also several times higher (4566 vs. 765 mg GAE/kg). For example, the fiber was characterized by a higher Mg content (990.88 vs. 222.02 mg/kg).

After regular drinking of chokeberry juice for 60 days, a significant increase in serum Cu concentration (from 0.955 mg/L to 0.999 mg/L), a significant increase in Mn (from 1.010 to 1.519 µg/L), as well as a significant increase in Se concentration (from 77.33 to 95.46 µg/L) were observed. An interesting observation is that drinking the juice did not cause a significant increase in Zn concentration, and even its level decreased, albeit insignificantly (Table 3).

The addition of fiber for the next 30 days caused a further increase in the tested parameters (Cu concentration increased from 0.999 to 1.004 mg/L) and Mn from 1.519 to 2.236 µg/L, stabilized the Se concentration (98.46 µg/L), and reduced the Zn concentration (to 1.019 mg/L) (Table 3).

We also assessed the effect of gender on the concentration of antioxidant elements in serum. We showed that gender did not significantly affect changes in the concentrations of Cu, Mn, Se, and Zn. Our studies indicate that men were characterized by a significantly higher median initial Zn concentration compared to women (1.122 vs. 1.015 mg/L).

The dietary intervention we carried out allowed us to state that the Cu/Zn ratio after 3 months of chokeberry juice consumption, including 1 month of additional fiber consumption, increased significantly to a value above 1 (from 0.980 to 1.018). Similarly, a significant increase was observed in women (from 1.013 to 1.047) and men (from 0.912 to 0.993) (Table 4).

After 3 months, we observed a positive statistically significant correlation between Zn concentration in plasma and Zn concentration in chokeberry juice (R = 0.219, *p* < 0.05) and a negative correlation between Zn concentration in plasma and Zn concentration in chokeberry fiber (R = −0.232, *p* < 0.05) (Table 5).

It was observed that the Cu/Zn molar ratio positively correlated with lower pulse and lower HDL–cholesterol value at the initial stage of analysis, before the intervention. Studies conducted after 60 days of juice consumption revealed a negative correlation between Cu/Zn and LDL–cholesterol concentration (correlation of results with data published in previous articles). After 90 days, a negative correlation was found between Cu/Zn and Mn and Se concentrations.

We also correlated previously published FRAP data with element concentrations described in this manuscript. We found a weak, negative, and significant correlation between the indicated parameter and Se concentration (R = −0.022, *p* < 0.05).

## 4. Discussion

So far, the impact of consuming chokeberry products on the status of trace elements in blood serum has not been assessed. As previously shown, chokeberry juice may be one of the sources of Cu and Mn (on average, 65.64 mg/kg in the case of Cu and 3.81 mg/kg in the case of Mn). In the case of Se and Zn, it provided smaller amounts (0.59 mg/kg for Zn and 15.79 µg/kg for Se). Chokeberry fiber assessed in the same study contained 36.13 mg/kg of Mn, 17.13 mg/kg of Zn, 6.17 mg/kg of Cu, and the least content of Se (61.03 µg/kg) [5]. Cu is a part of the enzyme ceruloplasmin, which catalyzes the oxidation of Fe in the body. This element also acted as a cofactor for some antioxidant enzymes (including superoxide dismutase, tyrosinase, and oxidases). The source of Cu in the diet will mainly be legumes, cereal products, nuts, and seeds [24,25]. Serum Cu levels among participants were within reference values before the start of the study. It changed statistically significantly after 2 and 3 months of intervention compared to the level before the intervention. As chokeberry products may be an important source of Cu in the diet, there may be concerns that the acceptable daily intake of Cu will be exceeded. However, it should be emphasized that Cu from plant products is absorbed to a lesser extent than from animal products [38]. The amount of Cu that we can absorb from food and water is relatively low. Additionally, the bioavailability of Cu from the diet ranges between 30 and 40% in industrialized countries, and the body has natural mechanisms that regulate its absorption [39]. Excess Cu leading to toxicity due to these regulatory mechanisms is relatively rare. The causes of excess Cu may be due to occupational and environmental exposure, adrenal dysfunction, liver disease, and the inborn errors of Cu metabolism. These factors may contribute to excessive Cu accumulation in tissues [40]. Cu toxicity may be caused by genetic disorders related to the absorption and excretion of Cu, such as Menkes disease and Wilson’s disease [41]. In turn, the clinical symptoms of Cu deficiency will be neurological disorders such as peripheral neuropathy and hematological disorders such as anemia, leukopenia, and thrombocytopenia. The causes of the deficiency may be a deficient diet, excessive Zn supplementation, previous gastrointestinal surgery, absorption problems, alcohol abuse, or long-term parenteral nutrition [42].

Significant changes were also noticed in the case of Mn, such as that the level increased significantly from 1.202 µg/L before the intervention to 2.341 µg/L after its completion. Mn levels among participants varied. It ranged from 0.116 to 3.502 µg/L before the intervention and from 0.343 to 6.831 µg/L. Such a significant difference may result from the different content of this ingredient in the diet; however, it should be emphasized that the Mn content in the serum does not always reflect the body’s actual resources. Humans absorb only about 1% to 5% of dietary Mn [43,44,45]. As dietary manganese absorption is low, it appears that other factors, such as overall manganese content in the diet among study participants, may have played a role. Mn in the body acts as a cofactor and a component of enzymes such as superoxide dismutase, pyruvate carboxylase, alkaline phosphatase, glucosyltransferase, and glutamine synthetase. Mn occurs in tea, nuts, legumes, cereal products, and seafood, as well as in smaller amounts in some fruits and vegetables [30,31]. Excess Mn is excreted mainly in the bile into the feces. In case of deficiency, its absorption increases [43,46]. Excessive Mn may contribute to so-called manganism, a neurodegenerative disorder associated with dopaminergic neuron death. The symptoms resemble Parkinson’s disease [47]. The main cause of excess in the body is occupational exposure, among miners, steel workers, and welders [48]. Mn deficiency is relatively rare. Deficiency symptoms include dermatitis, impaired hair and nail growth, increased serum calcium (Ca) and phosphorus (P) levels, decreased levels of cholesterol and coagulation proteins, and increased alkaline phosphatase activity [47,49].

In the case of Se, its serum level increased after the first stage, when participants consumed the juice, and then remained after the addition of fiber. Se occurs in the body in the form of selenoproteins. It is a component of some enzymes, such as glutathione peroxidase, deiodinase, selenoprotein-P, and thioredoxin reductase. It plays an important role in antioxidant defense, thyroid hormone production, DNA synthesis, and the body’s reproductive functions. It also participates in the proper functioning of the immune system by stimulating the formation of antibodies and the activity of T lymphocytes (helper and cytotoxic) and natural killer cells [50,51]. The sources of Se are both plant and animal products—nuts (especially Brazil nuts), seeds, cereal products, eggs, meat, milk, some vegetables, and fruits [52]. However, since chokeberry products do not provide a significant amount of Se, it is difficult to hypothesize that the main effect is the increase in serum Se [5]. In food, Se occurs mainly in the form of the compounds selenomethionine and selenocysteine. Se from the diet is relatively easily absorbed. Se homeostasis is maintained mainly through the excretion of excess in urine, and, in cases of higher intake, also in feces and through respiration [52,53]. Symptoms of excessive Se intake include a garlic odor from the mouth and a metallic taste in the mouth. Chronic excess of Se leads to selenosis, which manifests itself in hair loss, brittle nails, skin rash, nausea, diarrhea, fatigue, irritability, and nervous system disorders [52]. Se deficiency may result in decreased immunity and, therefore, increased susceptibility to infections. Long-term Se deficiency increases the risk of diseases such as Kaschin–Beck disease, Keshan disease, and cardiovascular diseases [54]. Se deficiency has been documented in countries such as New Zealand and China [55,56].

We observed that nutritional intervention did not increase serum Zn concentration. Zn is an element that has many different functions in the body, including antioxidant activity [57]. No effect on increasing Zn levels confirms the fact that chokeberry products are not a good source of Zn, as has been previously shown [5]. Excess Zn in the diet may cause symptoms such as Cu deficiency, anemia, or neutropenia. People who take excessive Zn supplementation and people with Wilson’s disease who require Zn supplementation as one of the treatment methods to control Cu levels in the body may be at risk of Zn toxicity. Additionally, people who come into contact with this element professionally are exposed to excess Zn due to the inhalation of Zn dust or fumes [58]. The decreased serum Zn concentration may be the result of both compensatory mechanisms and the fact that participants could not supplement with minerals during the intervention. Juice is not a source of Zn; therefore, its concentration decreased, which simultaneously translated into an incorrect Cu/Zn molar ratio in the serum. It is worth mentioning that men had higher baseline serum zinc levels than women (*p* < 0.05). Considering the larger number of women in the study, this may affect the generalizability of the study results.

In previous studies, the consumption of chokeberry products had a different effect on health depending on the duration of the study and the doses used [1]. It could be suggested that, in the case of minerals, this could also be important. Extending the time and amount of chokeberry juice consumption could translate into higher levels of the trace elements studied in the serum. In turn, increasing the supply of fiber could affect the decrease in the absorption of some minerals due to its known binding properties [59,60].

Chokeberry products (chokeberry juice and fiber) contain some trace elements; however, their consumption does not seem to have a key impact on the daily intake. It seems that chokeberry juice may be one of the sources of some trace elements; however, according to the current literature, fiber may hinder the absorption of elements from the diet, in particular, insoluble fiber fractions [59,60]. However, the studied chokeberry products can be considered one of the sources of trace elements; however, it is important to evaluate the bioavailability of these elements from chokeberry juice and fiber in future studies.

Despite obtaining interesting results, our study has some limitations. First, the number of women and men taking part in the study was not equal due to the difficulty of recruiting participants. Further studies should take into account an equal division of gender. Additionally, our study did not include a control group due to the difficulty in obtaining a product that would have the organoleptic characteristics of chokeberry juice and fiber but would not have other valuable ingredients, including ingredients responsible for the tart taste. Another limitation of the study is the fact that participants used their diet throughout the intervention. Future studies may be based, for example, on the use of a standardized diet in all participants to eliminate the influence of this factor. Our intervention lasted 90 days, which allows for the observation of short-term changes but does not allow for the assessment of the durability of long-term effects. Future studies should also focus on explaining the mechanism of the decrease in zinc concentration and assessing whether the addition of fiber has a beneficial effect on the level of this element in serum or limits its absorption. Finally, despite the control of participants during the study, the influence of confounding factors cannot be completely ruled out.

## 5. Conclusions

Our study has shown that the systematic consumption of chokeberry juice for 12 weeks, as well as the addition of chokeberry fiber to the diet, can increase the concentration of selected beneficial elements in the serum; however, this issue requires further research.

## Figures and Tables

**Table 1:** Average intake of antioxidant minerals, vitamins, and FRAP value in the diets during intervention.

Average Intake in Diet			
Antioxidant Minerals			
	**Stage 0**	**Stage 1**	**Stage 2**
Cu [mg/day]	1.36	1.41	1.39
Zn [mg/day]	11.02	11.15	11.01
Mn [mg/day]	1.96	1.92	1.87
Fe [mg/day]	13.94	13.83	13.98
Se [µg/day]	45.34	48.01	50.12
**Antioxidant Vitamins**			
	**Stage 0**	**Stage 1**	**Stage 2**
A [µg/day retinol and retinyl esters]	1089.62	1085.54	1088.02
β-karoten [µg/day]	3909.44	3918.5	3921.41
vitamin C [mg/day]	136.31	139.32	140.65
vitamin E [mg/day α-tocopherol equivalent]	10.73	10.61	10.72
FRAP value [mmol/kg]	16.78	16.80	16.81

**Table 2:** Composition of chokeberry juice and fiber used during dietary intervention.

Studied Component	Chokeberry Juice	Chokeberry Fiber
Content of Studied Component		
FRAP [mmol/kg]	97.41	6.51
TPC [mg GAE/kg]	4566	765
Total Flavonoids [mg QE/kg]	791.7	63.7
Total Anthocyanins [mg Cy-3-GL/kg]	257.5	26.11
Vitamin C [mg/kg]	112.89	56.03
Mg [mg/kg]	222.02	990.88
Fe [mg/kg]	1.07	60.314
Cu [mg/kg]	65.64	3.69
Mn [mg/kg]	6.49	38.61
Se [µg/kg]	26.86	212.91
Zn [mg/kg]	1.15	17.26
Hg [µg/kg]	0.28	3.40
Pb [µg/kg]	1.63	19.52
Cd [µg/kg]	1.95	14.64
As [µg/kg]	1.00	6.65
NO_2−_ [mg/kg]	4012	4.42
NO_3−_ [mg/kg]	38.63	41.41

FRAP—ferric reducing antioxidant power assay, GAE—gallic acid, QE—quercetin, Cy-3-GL—cyanidin-3-glucoside, NO_2−_—nitrite, NO_3−_—nitrate.

**Table 3:** Concentrations of antioxidant elements in serum depend on the test stage.

Parameter	Av. ± SD	Min.–Max.	Med.	Q1–Q3
	Total			
Cu (mg/L) stage 0	1.022 ± 0.143	0.804–1.616	0.995 ** 0/1, ** 0/2	0.912–1.105
stage 1	1.030 ± 0.142	0.800–1.609	0.999	0.921–1.110
stage 2	1.037 ± 0.175	0.810–1.970	1.004	0.923–1.109
Mn (µg/L) stage 0	1.202 ± 0.608	0.116–3.502	1.010 *** 0/1, *** 0/2	0.860–1.505
stage 1	1.696 ± 0.695	0.499–3.443	1.519 *** 1/2	1.161–0.930
stage 2	2.341 ± 1.018	0.343–6.831	2.236	1.571–2.917
Se (µg/L) stage 0	75.86 ± 16.82	31.46–118.34	77.33 *** 0/1, *** 0/2,	63.22–87.04
stage 1	100.17 ± 27.22	40.00–156.26	95.46	77.33–121.65
stage 2	99.51 ± 24.22	44.00–155.11	98.46	80.83–116.78
Zn (mg/L) stage 0	1.116 ± 0.340	0.746–3.120	1.060	0.953–1.164
stage 1	1.073 ± 0.205	0.662–1.875	1.041	0.981–1.137
stage 2	1.111 ± 0.325	0.738–2.747	1.019	0.930–1.153
	**Women**		**Men**	
**Parameter**	**Av. ± SD (Min.** **–Max.)**	**Med. (Q1–Q3)**	**Av. ± SD (Min.** **–Max.)**	**Med. (Q1–Q3)**
Cu (mg/L) stage 0	1.038 ± 0.150 (0.807–1.161)	1.006 (0.916–1.141)	0.991 ± 0.126 (0.804–1.229)	0.970 (0.897–1.088)
stage 1	1.045 ± 0.144 (0.821–1.609)	1.010 (0.940–1.144)	0.998 ± 0.134 (0.800–1.339)	0.974 (0.800–1.339)
stage 2	1.045 ± 0.143 (0.824–1.610)	1.011 (0.930–1.143)	1.030 ± 0.234 (0.810–1.970)	0.980 (0.892–1.081)
Mn (µg/L) stage 0	1.207 ± 0.652 (0.116–3.502)	1.008 (0.835–1.540)	1.193 ± 0.520 (0.373–2.623)	1.011 (0.860–1.372)
stage 1	1.703 ± 0.712 (0.499–3.443)	1.588 (1.145–2.091)	1.681 ± 0.673 (0.825–3.114)	1.311 (1.194–2.191)
stage 2	2.333 ± 1.054 (0.343–6.831)	2.227 (1.599–2.911)	2.358 ± 0.962 (1.132–4.198)	2.244 (1.541–3.248)
Se (µg/L) stage 0	74.80 ± 17.67 (31.46–118.34)	76.82 (59.41–86.88)	78.00 ± 15.02 (39.57–100.89)	81.29 (69.83–89.12)
stage 1	102.06 ± 27.17 (55.55–156.26)	98.49 (77.52–123.75)	96.32 ± 27.44 (40.00–156.11)	90.33 (75.69–117.44)
stage 2	101.14 ± 24.44 (52.42–155.11)	99.27 (86.07–120.95)	96.21 ± 23.89 (44.00–145.33)	94.13 (80.71–115.59)
Zn (mg/L) stage 0	1.097 ± 0.392 (0.770–3.120)	1.015 (0.933–1.113) **	1.155 ± 0.195 (0.764–1.603)	1.122 (1.042–1.269) **
stage 1	1.057 ± 0.206 (0.662–1.875)	1.020 (0.974–1.121)	1.105 ± 0.201 (0.788–1.684)	1.055 (0.998–1.184)
stage 2	1.117 ± 0.355 (0.738–2.747)	0.993 (0.913–1.153)	1.100 ± 0.258 (0.811–2.072)	1.026 (0.984–1.157)

Av.—average, Max.—maximum, Med.—median, Min.—minimum, Q1—lower quartiles, Q3—upper quartiles, SD—standard deviation. ** *p* < 0.01, *** *p* < 0.001.

**Table 4:** Cu/Zn molar ratio values for individual test stages.

**Parameter**	**Group**	**Av. ± SD (Min.–Max.)**	**Med. (Q1–Q3)**
Cu/Zn—stage 0	Total	0.993 ± 0.245 (0.346–1.707)	0.980 (0.832–1.110) *
Cu/Zn—stage 1		1.020 ± 0.226 (0.508–1.703)	0.993 (0.906–1.125)
Cu/Zn—stage 2		1.022 ± 0.279 (0.341–1.956)	1.018 (0.857–1.201) *
Cu/Zn—stage 0	Women	1.033 ± 0.251 (0.346–1.707)	1.013 (0.863–1.200) *
Cu/Zn—stage 1		1.050 ± 0.228 (0.513–1.703)	1.000 (0.923–1.144)
Cu/Zn—stage 2		1.030 ± 0.270 (0.341–1.793)	1.047 (0.857–1.234) *
Cu/Zn—stage 0	Men	0.911 ± 0.213 (0.540–1.590)	0.912 (0.785–1.040) *
Cu/Zn—stage 1		0.958 ± 0.212 (0.507–1.543)	0.952 (0.832–1.052)
Cu/Zn—stage 2		1.005 ± 0.301 (0.487–1.956)	0.993 (0.847–1.074) *

Av.—average, Max.—maximum, Med.—median, Min.—minimum, Q1—lower quartiles, Q3—upper quartiles, SD—standard deviation, * *p* < 0.05.

**Table 5:** Correlations between the content of elements in plasma and the content of elements in chokeberry juice and fibre at individual stages of the study (* *p* < 0.05).

Parameter	Stage 1		Stage 2	
	R	*p*	R	*p*
Cu in plasma/Cu in chokeberry juice	0.16	0.16	0.15	0.18
Mn in plasma/Mn in chokeberry juice	0.04	0.73	0.03	0.73
Se in plasma/Se in chokeberry juice	−0.06	0.57	−0.06	0.61
Zn in plasma/Zn in chokeberry juice	0.17	0.12	0.22	0.05 *
Cu in plasma/Cu in chokeberry fiber	na	na	0.14	0.20
Mn in plasma/Mn in chokeberry fiber	na	na	0.03	0.79
Se in plasma/Se in chokeberry fiber	na	na	0.06	0.62
Zn in plasma/Zn in chokeberry fiber	na	na	−0.23	0.04 *

na—not applicable.

## Data Availability

All data are available upon request from the corresponding author.
